# ﻿A new species of *Ptychognathus* Stimpson, 1858, from East and Southeast Asia, previously identified as *Ptychognathusbarbatus* (A. Milne-Edwards, 1873) (Crustacea, Decapoda, Brachyura, Varunidae)

**DOI:** 10.3897/zookeys.1243.152263

**Published:** 2025-06-27

**Authors:** Jhih-Wei Hsu, Jose Christopher E. Mendoza, Hsi-Te Shih

**Affiliations:** 1 Department of Life Science, National Chung Hsing University, Taichung 402, Taiwan; 2 Lee Kong Chian Natural History Museum, Faculty of Science, National University of Singapore, 2 Conservatory Drive, 117377 Singapore, Singapore; 3 Global Change Biology Research Center, National Chung Hsing University, Taichung 402, Taiwan

**Keywords:** description, mitochondrial cytochrome c oxidase subunit I, morphology, *Ptychognathusdajie* sp. nov.

## Abstract

The brackish-water crabs of the genus *Ptychognathus* Stimpson, 1858, currently the most diverse genus within the family Varunidae, typically inhabit estuaries or seashores influenced by freshwater. Among the species in this genus, a commonly recorded species identified as *Ptychognathusbarbatus* (A. Milne-Edwards, 1873) has been recorded across the western Pacific, including Japan, Taiwan, China, the Philippines, Malaysia, Indonesia, and New Caledonia (type locality). However, some previous studies suggested that most of these records do not represent the true *P.barbatus*. The present study reexamines these records and specimens and describes a new species, *P.dajie***sp. nov.**, from East and Southeast Asia, based on morphological differences and genetic data from mitochondrial cytochrome c oxidase subunit 1 (COI). This new species is similar to its congeners but can be distinguished by the features of the carapace, the setation of ambulatory legs, and male first gonopods. In addition, the intraspecific distances of *P.dajie***sp. nov.** are below 0.77%, while the interspecific distances between this new species and other species are larger than 11.89%.

## ﻿Introduction

*Ptychognathus* Stimpson, 1858, is currently the most speciose genus within the family Varunidae H. Milne Edwards, 1853, with a wide distribution across the Indo-West Pacific. To date, a total of 32 species of *Ptychognathus* have been described (Sasaki 2023; Hsu and Shih 2024a; De Mazancourt et al. 2024; Patel et al. 2025). Although the genus has a broad distribution, most species are distributed in the western Pacific region (Hsu et al. 2022; Sasaki 2023; De Mazancourt et al. 2024); and several recently described species of *Ptychognathus* are also from this area (e.g., *P.lipkei* NK Ng, 2010; *P.makii* Hsu & Shih, 2020; *P.stimpsoni* Hsu & Shih, 2020; and *P.sakaii* Hsu, Shih & Li, 2022). In this highly diverse region, some studies suggested that there are still undescribed species of this genus (Hsu and Shih 2020).

*Ptychognathusbarbatus* (A. Milne-Edwards, 1873) was originally described as *Gnathograpsusbarbatus* A. Milne-Edwards, 1873, based on specimens from New Caledonia (see A. Milne-Edwards 1873). This species was subsequently recorded in several localities, including many areas of East and Southeast Asia, such as China, Japan, Taiwan, Malaysia, and Indonesia (De Man 1895, 1902; Sakai 1939, 1976; Dai et al. 1986; Dai and Yang 1991; Sasaki 2023). Recent studies (Hsu and Shih 2020; Hsu et al. 2022), however, have indicated that many of the records of “*Ptychognathusbarbatus*” from East and Southeast Asia do not represent the true *P.barbatus*, but rather, is an undescribed species (see Ng 2006). In her unpublished doctoral thesis, Ng (2006) undertook a systematic revision of the entire family Varunidae, including the genus *Ptychognathus*. Therein she established a new genus, “*Cognatus*”, under which she included two new species from East and Southeast Asia, viz. “*Cognatuscavaterminus*”, from China, Japan, Taiwan, Philippines, Indonesia, Malaysia, and “*C.benokiensis*”, from the Ryukyu Islands (Ng 2006: 384). As the taxonomic and nomenclatural acts in NK Ng’s thesis were indicated as unpublished (see Disclaimer in thesis, p. xiii), the names of these new taxa are considered unavailable in accordance with Articles 8 and 10 of the International Code of Zoological Nomenclature (ICZN 1999). For the present study, an opportunity to validate and update the work of Ng (2006) work has presented itself. Examination of her material, as well as additional material from East and Southeast Asia previously identified as *P.barbatus*, and topotypic material of *P.barbatus* sensu stricto, confirms that the relevant records of “*P.barbatus*” from the region and the species placed in “*Cognatus*” by NK Ng have the sufficient morphological and molecular distinctions to be recognized as a new species of *Ptychognathus*.

In recent years, several studies on *Ptychognathus* have used molecular analyses to assist in species differentiation, demonstrating that the COI (cytochrome *c* oxidase subunit I) gene is useful for the classification and identification of most *Ptychognathus* species. This marker has also been effective in confirming more significant intraspecific morphological variation in some species (Hsu and Shih 2020, 2024a; De Mazancourt et al. 2024). Therefore, present study used COI to aid in the identification of this new species and to check intraspecific and interspecific morphological variation.

## ﻿Materials and methods

**Figure 1.**
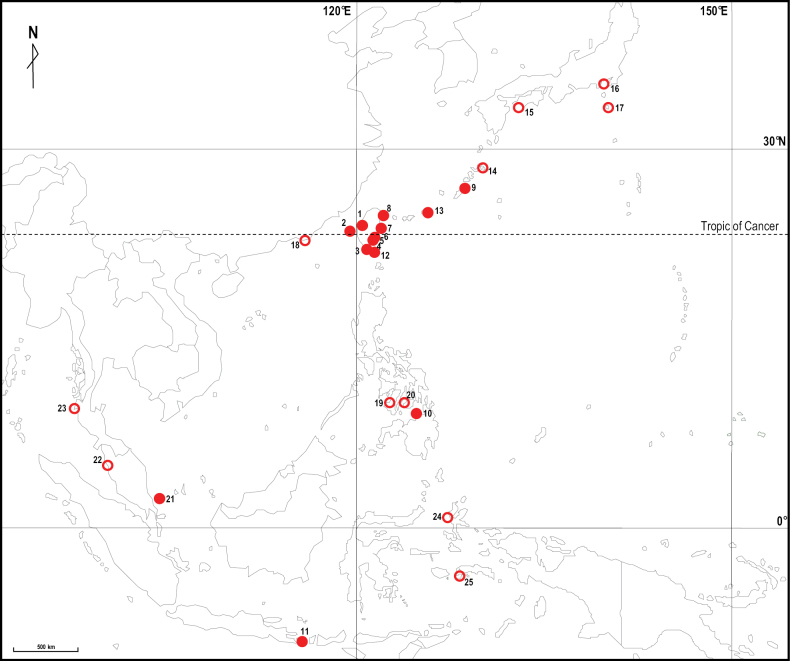
The collection sites of *Ptychognathusdajie* sp. nov. in East and Southeast Asia. Solid symbols mean collection sites of specimens used in present study; empty symbols mean the additional records only from references. 1–11 refer to Table 1; 12, Lanyu Island, Taiwan; 13, Miyako Islands, Japan; 14, Amami Island, Japan; 15, Kochi and Ehime, Japan; 16, Kanagawa, Japan; 17, Izu Islands, Japan; 18, Guangdong, China; 19, Negros, the Philippines; 20, Cebu, the Philippines; 21, Tioman Island, Malaysia; 22, Penang, Malaysia; 23, Phuket, Thailand; 24, Ternate, Indonesia; 25, Ambon Island, Indonesia.

The examined specimens are deposited in the Zoological Collections of the Department of Life Science, National Chung Hsing University, Taichung, Taiwan (**NCHUZOOL**), and the Zoological Reference Collection of the Lee Kong Chian Natural History Museum, National University of Singapore (**ZRC**). The collection sites of the specimens are shown in Fig. 1.

The following abbreviations are used in present study: **CW** = carapace width, **CL** = carapace length; **P2–P5** = first to fourth ambulatory legs; **G1** = male first gonopod; **G2** = male second gonopod; **ovig.** = ovigerous.

Genomic DNA was isolated from the muscle tissue of legs, gills, or eggs by using the GeneMark tissue and cell genomic DNA purification kit (Taichung, Taiwan). A portion of the COI gene was amplified with PCR using the primers LCO1490 (5’-GGTCAACAAATCATAAAGATATTGG-3’), HCO2198 (5’-TAAACTTCAGGGTGACCAAAAAATCA-3’) (Folmer et al. 1994), LCOB (5’-CAAAYCATAAAGAYATYGG-3’), and HCOex3 (5’-GCTCANACTACRAATCCT A-3’) (Shih et al. 2022). The PCR conditions for the above primers were denaturation for 50 s at 94 °C, annealing for 70 s at 45–47 °C, and extension for 60 s at 72 °C (40 cycles), followed by extension for 10 min at 72 °C. Amplicon sequences were obtained using an automated 3730 Series DNA Analyzer (Applied Biosystems, Foster City, CA, USA) after verification with the complementary strand. Haplotype sequences have been deposited in the GenBank database, and their accession numbers are listed in Table 1. The molecular analyses also included other haplotypes from GenBank published in Hsu and Shih (2020, 2024a, 2024b), Winarni et al. (2023), and De Mazancourt et al. (2024).

**Table 1.** The haplotypes of the cytochrome c oxidase subunit I (COI) for the specimens of *Ptychognathus* species used in present study. The numbers in parentheses following the localities indicate the collection sites, corresponding to those shown in Fig. 1.

Species	Locality	Sample size	Accession number	References
*P.dajie* sp. nov.	Taiwan: Fubao, Changhua [1]	1	MW000767	Hsu and Shih (2020)
	Taiwan: Watong, Baisha, Penghu [2]	1	MW000768	Hsu and Shih (2020)
	Taiwan: Baoli R. estuary, Pingtung [3]	1	MW000768	Hsu and Shih (2020)
	Taiwan: Dulanwan, Taitung [4]	1	MW000767	Hsu and Shih (2020)
	Taiwan: Shanyuan, Taitung [5]	1	MW000768	Hsu and Shih (2020)
	Taiwan: Shanyuan, Taitung [5]	1	MW000767	Hsu and Shih (2020)
	Taiwan: Jihuei, Taitung [6]	1	MW000767	Hsu and Shih (2020)
	Taiwan: Yanliao, Hualien [7]	1	MW000769	Hsu and Shih (2020)
	Taiwan: Dasi R. estuary, Yilan [8]	1	MW000767	Hsu and Shih (2020)
	Japan: Benoki, Okinawa Island [9]	1	PV557377	present study
	Philippines: Camiguin Island [10]	1	PV557378	present study
	Philippines: Camiguin Island [10]	1	MW000770	present study
	Indonesia: Bali [11]	1	MW000770	Hsu and Shih (2020)
*P.guijulugani*	Indonesia: Bali	1	PV557376	present study
*P.pusillus*	Australia: Dolly Beach, Christmas Island	1	PP294706	Hsu and Shih (2024a)
*P.amikee*	Vanuatu: Kirilou River, Gaua Island	1	PP294699	Hsu and Shih (2024a)
	Vanuatu: Kirilou River, Gaua Island	1	PP294700	Hsu and Shih (2024a)
*P.takahasii*	Taiwan: Jihuei, Taitung	1	MW000786	Hsu and Shih (2020)
*P.insolitus*	Taiwan: Houwan, Pingtung	1	MW000774	Hsu and Shih (2020)
	Taiwan: Houwan, Pingtung	1	MW000775	Hsu and Shih (2020)
*P.similis*	Wallis & Futuna (France): Futuna Island	1	OR864756	De Mazancourt et al. (2024)
	Wallis & Futuna (France): Futuna Island	1	OR864757	De Mazancourt et al. (2024)
*P.hachijoensis*	Taiwan: Chaishan, Kaohsiung	1	MW000771	Hsu and Shih (2020)
	Taiwan: Houwan, Pingtung	1	MW000772	Hsu and Shih (2020)
	Taiwan: Jioupeng, Pingtung	1	MW000773	Hsu and Shih (2020)
*P.ngankeeae*	Wallis & Futuna (France): Futuna Island	1	OR864752	De Mazancourt et al. (2024)
	Wallis & Futuna (France): Futuna Island	1	OR864753	De Mazancourt et al. (2024)
*P.barbatus*	New Caledonia: Grande Terre Island	1	OR864745	De Mazancourt et al. (2024)
*P.stimpsoni*	Taiwan: Wanlitong, Pingtung	1	MW000782	Hsu and Shih (2020)
	Philippines: Camiguin	1	MW000784	Hsu and Shih (2020)
	Philippines: Camiguin	1	MW000783	Hsu and Shih (2020)
	Philippines: Camiguin	1	MW000785	Hsu and Shih (2020)
*P.sakaii*	Taiwan: Dingtanzih, Pingtung	2	MW000787	Hsu and Shih (2020)
*P.lipkei*	Taiwan: Yeyin, Lanyu Island	1	PP294702	Hsu and Shih (2024a)
	Taiwan: Yeyin, Lanyu Island	1	PP294703	Hsu and Shih (2024a)
	Taiwan: Yeyin, Lanyu Island	1	PP294704	Hsu and Shih (2024a)
*P.glaber*	Japan: Ogasawara (Bonin)Islands	1	PP272962	Hsu and Shih (2024b)
*P.ishii*	Taiwan: Dulanwan, Taitung	1	MW000778	Hsu and Shih (2020)
	Taiwan: Gangkou R. estuary, Pingtung	1	MW000776	Hsu and Shih (2020)
	Taiwan: Gangkou R. estuary, Pingtung	1	MW000777	Hsu and Shih (2020)
*P.riedelii*	Philippines: Cebu Island	1	PP294705	Hsu and Shih (2024a)
	Philippines: Cebu Island	1	PP272963	Hsu and Shih (2024b)
*P.pilosus*	Taiwan: Gangkou R. estuary, Pingtung	1	MW000781	Hsu and Shih (2020)
*P.altimanus*	Taiwan: Gangkou R. estuary, Pingtung	1	MW000764	Hsu and Shih (2020)
	Taiwan: Linbian R. estuary, Pingtung	1	MW000765	Hsu and Shih (2020)
	Indonesia: Cilacap, Central Java	1	OQ852797	Winarni et al. (2023)
	Taiwan: Gangkou R. estuary, Pingtung	1	MW000763	Hsu and Shih (2020)
	Taiwan: Linbian R. estuary, Pingtung	1	MW000766	Hsu and Shih (2020)
*P.makii*	Taiwan: Gangkou R. estuary, Pingtung	1	MW000779	Hsu and Shih (2020)
	Taiwan: Gangkou R. estuary, Pingtung	1	MW000780	Hsu and Shih (2020)
*P.crassimanus*	French Polynesia: Fatu Hiva Island	1	OR864744	De Mazancourt et al. (2024)
	French Polynesia: Fatu Hiva Island	1	OR864760	De Mazancourt et al. (2024)
	French Polynesia: Fatu Hiva Island	1	OR864759	De Mazancourt et al. (2024)
	French Polynesia: Fatu Hiva Island	1	OR864751	De Mazancourt et al. (2024)
	French Polynesia: Fatu Hiva Island	1	OR864742	De Mazancourt et al. (2024)
	French Polynesia: Fatu Hiva Island	1	OR864750	De Mazancourt et al. (2024)
*P.easteranus*	French Polynesia: Tahiti	1	OR864766	De Mazancourt et al. (2024)
	French Polynesia: Tahiti	1	OR864767	De Mazancourt et al. (2024)
	French Polynesia: Mangareva Island	1	OR864763	De Mazancourt et al. (2024)
	French Polynesia: Rapa Island	1	OR864755	De Mazancourt et al. (2024)
	French Polynesia: Rapa Island	1	OR864765	De Mazancourt et al. (2024)
	French Polynesia: Tahiti	1	OR864748	De Mazancourt et al. (2024)
	French Polynesia: Rurutu Island	1	OR864746	De Mazancourt et al. (2024)
	French Polynesia: Tahiti	1	OR864749	De Mazancourt et al. (2024)
	French Polynesia: Mangareva Island	1	OR864762	De Mazancourt et al. (2024)
	French Polynesia: Rapa Island	1	OR864764	De Mazancourt et al. (2024)
	French Polynesia: Rapa Island	1	OR864754	De Mazancourt et al. (2024)
	French Polynesia: Rapa Island	1	OR864761	De Mazancourt et al. (2024)

A neighbor-joining (NJ) tree for COI sequences was constructed using the Kimura (1980) 2-parameter (K2P) model with the 2,000 bootstrap reiterations and complete deletion option in MEGA (v. 11, Tamura et al. 2021). Pairwise distance estimates based on the K2P model, using the pairwise deletion option, were calculated in MEGA to assess interand intraspecific genetic diversity between the new species and other species of *Ptychognathus*. Due to the large number of species, and the considerable morphological differences between some of them and the new species, interspecific genetic diversities were calculated only by comparing the new species with morphologically similar species.

## ﻿Results

### ﻿Taxonomy

**Family Varunidae H. Milne Edwards, 1853**

**Subfamily Varuninae H. Milne Edwards, 1853**

**Genus *Ptychognathus* Stimpson, 1858**

#### ﻿ Ptychognathus dajie sp. nov.

Taxon classificationAnimaliaDecapodaVarunidae

67771559-2D20-584D-963D-39C8EC711AE2

https://zoobank.org/4A93B11E-2674-47B4-BEFA-2137407439C2

Figs 2 , 3 , 5A

Ptychognathus barbatus – De Man 1895: 105, fig. 23 [Indonesia: Atjeh and Ambon Island; Malaysia: Penang] (part); De Man 1898: 698, fig. 23 [Indonesia: Atjeh; Malaysia: Penang] (part); De Man 1902: 505 [Indonesia: Ternate]; Tesch 1918: 87 (key) (part?); Maki and Tsuchiya 1923: 188 [Taiwan]; Tweedie 1936: 50 [Malaysia: Tioman Island]; Sakai 1939: 659, text-fig. 113 [Japan: Ryukyu Islands]; Lin 1949: 28 [Taiwan]; Sakai 1976: 638, text-fig. 348, pl. 219(2) [Japan: Ryukyu Islands]; Dai et al. 1986: 468, fig. 262, pl. 65(8) [China: Guangdong]; Fukui et al. 1989: 229, fig. 14 [Taiwan]; Dai and Yang 1991: 515, fig. 262, pl. 65(8) [China: Guangdong]; Wang and Liu 1996a: 109, figs 136–137 [Taiwan]; Wang and Liu 1996b: 77, 2 unnumb. figs [Taiwan]; Jeng 1998: 123 [Taiwan]; Muraoka 1998: 52 [Japan; Taiwan]; Kato and Okuno 2001: 139, 1 unnumb. fig. [Japan: Izu Islands]; Lee 2001: 130, 1 unnumb. fig. [Taiwan]; Kishino et al. 2001: 126 [Japan: Amami Island]; Ng et al. 2001: 46 [Taiwan] (list); Akio and Yoshihiko 2007 [Japan: Kochi and Ehime Prefectures]; Ng et al. 2008: 228 (list) (part); Naruse 2010: 54 [Japan: Okinawa Island]; Ng et al. 2017: 115 [Taiwan] (list); Toyota et al. 2019: 298, 5 unnumb. figs [Japan: Kanagawa, Ryukyu Islands, Izu Islands]; Wada 2021: 107 [Taiwan]; Sasaki 2023: 15534 (list) (part). non Ptychognathusbarbatus A. Milne-Edwards, 1873.
Ptychognathus aff. barbatus – Hsu and Shih 2020: 7, tab. 1 [Taiwan; Indonesia: Bali Island]; Hsu et al. 2022: 8 [Taiwan] (key); Hsu and Shih 2024a: 160, tab. 2 [Taiwan].
Cognatus cavaterminus NK Ng, 2006: 387, figs 107, 108 (unpublished MS name), nomen nudum.
Cognatus benokiensis NK Ng, 2006: 392 (unpublished MS name), nomen nudum.

##### Type material.

***Holotype*.** Malaysia • 1 male (13.2 × 11.6 mm); Pahang, Tioman Island, Paya River; 7–12 Sep. 2002; ZRC 2024.0072. ***Paratypes*.** Malaysia • 19 males (14.8 × 13.0–6.9 × 6.1 mm), 22 females (10.9 × 9.4–5.2 × 4.1 mm), 2 ovig. females (8.9 × 7.6, 7.3 × 7.0 mm); same as the holotype; 7–12 Sep. 2002; ZRC 1985.1569–1573. 2 males (14.8 × 13.0, 14.3 × 12.3 mm), 3 females (10.9 × 9.4, 10.6 × 9.2, 10.5 × 9.1 mm); same as the holotype; 7–12 Sep. 2002; NCHUZOOL 17356.

##### Other material.

**Japan** • 9 males (15.5 × 13.8–9.5 × 8.8 mm), 4 females (12.0 × 11.0–9.0 × 8.4 mm), 1 ovig. female (10.8 × 9.7 mm); Ryukyu Islands, Okinawa, Miyako, Urasoko; 11 Apr. 2002; NCHUZOOL 17335. 28 males (10.0 × 8.9–4.5 × 4.0 mm); Ryukyu Islands, Okinawa, Benoki, Benoki River; 7 Jul. 1990; ZRC 2008.0035 (labeled as “*Cognatusbenokiensis*” in NK Ng 2006’s dissertation). **Taiwan** • 10 males (12.4 × 10.9–7.7 × 6.8 mm), 5 females (15.3 × 13.1–8.9 × 8.0 mm); Yilan, Toucheng, Dasi; 15 Aug. 2016; NCHUZOOL 16063. 2 males (14.7 × 12.9, 10.1 × 9.0 mm), 1 female (13.0 × 11.0 mm), 1 ovig. female (13.2 × 11.3 mm); Hualien, Shoufeng, Yanliao; 29 Jun. 2016; NCHUZOOL 16064. 2 females (7.4 × 6.5, 6.7 × 5.7 mm); Taitung, Donghe, Dulanwan; 9 Aug. 2017; NCHUZOOL 16065. 1 male (13.0 × 11.1 mm); Taitung, Donghe, Dulanwan; 27 Jul. 2014; NCHUZOOL 16066. 2 males (11.0 × 10.1, 11.0 × 10.0 mm), 1 female (9.4 × 8.3 mm); Taitung, Chenggong, Gihui; 28 Apr. 2017; NCHUZOOL 16067. 3 males (14.9 × 12.7–12.1 × 10.8 mm); Changhua, Fubao; 16 Jan. 2017; NCHUZOOL 16075. 2 males (20.2 × 16.8, 18.9 × 16.2 mm), 1 female (11.5 × 10.3 mm); Kaohsiung, Linyuan, Gaoping R. estuary; 29 Apr. 2009; NCHUZOOL 16074. 4 males (16.9 × 14.7–9.9 × 8.9 mm), 1 female (8.6 × 7.7 mm); Pingtung, Hengchun, Houwan; 11 Jul. 2017; NCHUZOOL 16073. 6 males (10.1 × 9.0–5.7 × 5.0 mm), 1 female (10.0 × 9.1 mm), 1 ovig. female (11.0 × 10.0 mm); Pingtung, Hengchun, Houwan; 22 Sep. 2021; NCHUZOOL 17343. 1 male (13.0 × 11.4 mm); Pingtung, Checheng, Baoli R. estuary; 23 June 2014; NCHUZOOL 16072. 8 males (13.1 × 11.3–9.3 × 8.2 mm), 3 females (11.9 × 10.6–8.8 × 7.7 mm), 2 ovig. females (9.7 × 8.7, 8.2 × 7.1 mm); Pingtung, Hengchun, Wanlitong; 11 Dec. 2018; NCHUZOOL 16070. 3 males (13.9 × 12.0–12.6 × 10.9 mm), 1 female (9.0 × 8.1 mm), 1 ovig. female (11.1 × 9.6 mm); Pingtung, Hengchun, Wanlitong; 13 Mar. 2021; ZRC 2022.0839. 15 males (13.0 × 11.4–7.8 × 7.1 mm), 4 females (13.6 × 11.7–9.3 × 8.4 mm), 2 ovig. females (12.5 × 10.9, 7.7 × 7.0 mm); Pingtung, Hengchun, Wanlitong; 13 Mar. 2021; NCHUZOOL 17342. 5 males (11.4 × 10.2–7.8 × 7.1 mm), 1 female (9.4 × 8.4 mm); Pingtung, Hengchun, Dingtanzih; 19 Mar. 2018; NCHUZOOL 16071. 3 males (10.5 × 9.4–6.5 × 5.8 mm), 1 female (8.9 × 7.9 mm); Pingtung, Hengchun, Gangkou R. estuary; 18 Aug. 2016; NCHUZOOL 16069. 1 male (14.7 × 12.8 mm); Pingtung, Manjhou, Jhonggang R. estuary; 7 Nov. 2018; NCHUZOOL 16068. 2 males (14.0 × 12.3, 7.0 × 6.3 mm), 1 female (10.8 × 9.7 mm); Penghu Islands, Baisha, Watong; 2 Sep. 2014; NCHUZOOL 16076. 2 males (14.5 × 12.8, 9.3 × 8.6 mm), 2 females (14.2 × 12.7, 11.3 × 10.3 mm); Lanyu Island, Yeyou; 13 Mar. 2023; NCHUZOOL 17341. **Philippines** • 5 males (15.3 × 13.7–11.2 × 10.2 mm), 1 female (14.5 × 12.6 mm), 2 ovig. females (16.8 × 14.5, 13.6 × 12.1 mm); Camiguin Island; 30 Aug. 2003; NCHUZOOL 17336. **Indonesia** • 1 ovig. female (8.1 × 7.2 mm); Bali Island; 18 Jul. 2014; NCHUZOOL 16504.

##### Comparative material.

*Ptychognathusamikee* Hsu & Shih, 2024: VANUATU • 1 male (holotype, 15.8 × 13.6 mm); Gaua Island, Kirilou River; 18 Jul. 2005; MNHN-B29852. 1 female (paratype, 17.4 × 14.6 mm), Gaua Island, Lembot River; 20 July 2005; MNHN-B29853. *Ptychognathusbarbatus* (A. Milne-Edwards, 1873): FRANCE • 4 males (10.2 × 8.5–8.6 × 7.3 mm), 3 females (8.5 × 6.9–7.0 × 6.1 mm); New Caledonia; 8 Nov. 2006; ZRC 2019.1806. *Ptychognathusglaber* Stimpson, 1858: Japan • 2 males (11.4 × 9.5, 10.6 × 8.6 mm), 2 females (11.4 × 9.6, 11.0 × 9.1 mm); Ogasawara Islands, Chichi-jima Island, Futami, Kiyose River; 10 Dec. 2005; ZRC 2023.0086. *Ptychognathusguijulugani* Rathbun, 1914: Indonesia • 14 males (9.1 × 7.9–6.6 × 5.8 mm), 6 females (8.9 × 7.4–5.9 × 5.2 mm) 2 ovig. females (7.2 × 6.3, 6.9 × 6.2 mm); Bali Island; 18 Jul. 2014; NCHUZOOL 17334. *Ptychognathushachijoensis* Sakai, 1955: Taiwan • 4 males (9.8 × 7.7–7.0 × 5.9 mm), 6 females (8.0 × 6.7–6.3 × 5.4 mm); Hualien, Shoufeng, Yanliao; 29 Jun. 2016; NCHUZOOL 15809. *Ptychognathusinsolitus* Osawa & NK Ng, 2006: Taiwan • 1 male (9.1 × 6.3 mm), 2 females (7.9 × 6.0, 5.8 × 4.6 mm); Taitung, Donghe, Dulanwan; 29 Jun. 2016; NCHUZOOL 16041. *Ptychognathusishii* Sakai, 1939: Taiwan • 2 males (11.4 × 8.8, 8.9 × 7.3 mm), 2 females (9.1 × 8.6, 8.9 × 7.1 mm); Yilan, Toucheng, Dasi; 16 Aug. 2016; NCHUZOOL 16034. *Ptychognathuslipkei* NK Ng, 2010: Philippines • 4 males (paratypes, 14.6 × 11.6–11.0 × 9.2 mm); Cebu Island, Matutinao, Matutinao River mouth, Kawasan Falls; 30 Jul. 2003; ZRC 2013.1823. *Ptychognathuspusillus* Heller, 1865: AUSTRALIA • 2 males (13.2 × 11.3, 10.5 × 9.2 mm), 2 females (14.2 × 12.0, 13.2 × 11.3 mm); Christmas Island, Dolly Beach; 29 Jan. 2010; ZRC 2023.1844. *Ptychognathussakaii* Hsu, Shih & Li, 2022: Taiwan • 1 male (holotype, 6.7 × 5.5 mm); Pingtung, Hengchun, Dingtanzih; 3 Apr. 2019; NCHUZOOL 17047. 3 males (paratypes, 6.7 × 5.5–5.7 × 5.0 mm), 1 ovig. female (5.9 × 5.2 mm); Pingtung, Hengchun, Dingtanzih; 3 Apr. 2019; NCHUZOOL 16503. *Ptychognathusstimpsoni* Hsu & Shih, 2020: Taiwan • 1 male (holotype, 7.9 × 6.6 mm); Pingtung, Hengchun, Wanlitong; 15 Aug. 2016; NCHUZOOL 16501. Philippines • 16 males (paratypes, 10.9 × 9.0–7.3 × 6.2 mm), 4 females (8.8 × 7.2–7.5 × 6.3 mm); Camiguin Island; 31 Aug. 2003; NCHUZOOL 16502. *Ptychognathustakahasii* Sakai, 1939: Taiwan • 4 males (10.1 × 8.6–8.9 × 7.8 mm), 2 females (9.7 × 8.5, 7.0 × 6.3 mm), 2 ovig. females (8.9 × 7.7, 8.0 × 6.9 mm); Taitung, Chenggong, Gihui; 28 Apr. 2017; NCHUZOOL 16058.

##### Diagnosis.

**Figure 2.**
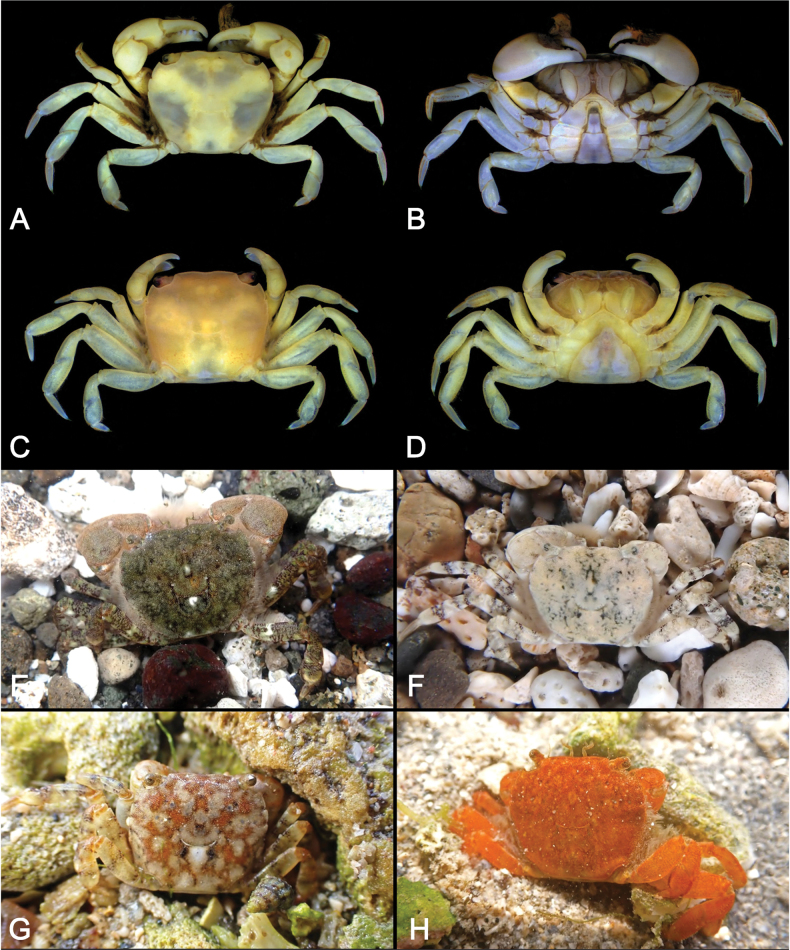
*Ptychognathusdajie* sp. nov. **A, B.** Holotype male (13.2 × 11.6 mm, ZRC 2024.0072); **C, D.** Paratype female (10.6 × 9.2 mm, NCHUZOOL 17356); **E.** Male (NCHUZOOL 17341); **F.** Male (NCHUZOOL 17343); **G, H.** Males (NCHUZOOL 17342). **A, C.** Dorsal view; **B, D.** Ventral view; **A–D.** Preserved specimens; **E–H.** Color in life.

**Figure 3.**
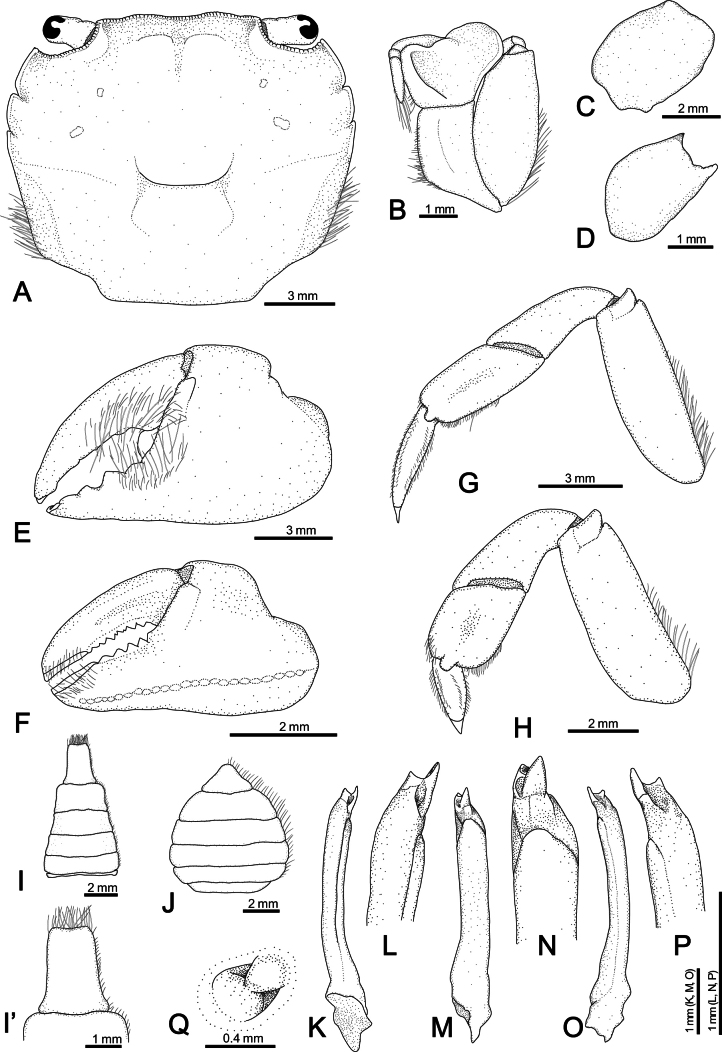
*Ptychognathusdajie* sp. nov. **A–C, E, G–I, K–P.** Holotype male (13.2 × 11.6 mm, ZRC 2024.0072); **D, F, J, Q.** Paratype female (10.6 × 9.2 mm, NCHUZOOL 17356). **A.** Carapace; **B.** Left third maxilliped; **C, D.** Left carpus of cheliped (dorsal view); **E, F.** Outer view of left cheliped; **G.** left third ambulatory leg (P4); **H.** Left forth ambulatory leg (P5); **I, J.** Pleon; **I’.** telson; **K, L.** Right G1 (dorsal view); **M, N.** Right G1 (lateral view); **O, P.** Right G1 (ventral view); **Q.** Right vulva.

Carapace (Figs 2A, C, 3A) subquadrate (CW/CL ~ 1.1–1.2); dorsal surface glabrous. Front broad, frontal margin concave medially, slightly divided into two indistinct lobes; postfrontal region distinct, separated into two lobes by shallow grooves. Anterolateral margin with three teeth including orbital tooth, third tooth small but distinct. Exopod of third maxilliped (Figs 2B, D, 3B) ~ 1.3 × as wide as ischium. Chelipeds (Fig. 2) symmetrical; inner angle of male carpus (Fig. 3C) without spines; inner surface of palm glabrous; in male (Fig. 3E), proximal 1/2 of fingers with tufts of long dense soft setae on outer surface, slightly extending to palm side; base of fingers with pulvinus in male; setae and pulvinus absent in female (Fig. 3F). P4 (Figs 3G, 5A) merus densely invested with long setae on proximal 1/2–2/3 of anterior margin; propodus densely invested with short setae on distal 1/3–1/2 of posterior margin and of distal anterior margin, with sparsely scattered long setae; dactylus densely invested with short setae on most of anterior and posterior margins. P5 (Figs 3H, 5A) merus with densely covered with long setae on proximal 2/3–3/4 of anterior margin; propodus densely covered with short setae on distal 1/3–1/2 of posterior margin and distal anterior margin, with sparsely scattered long setae; dactylus densely invested with short setae on almost entire anterior and posterior margins, denser on anterior margin. Male pleon (Fig. 3I) narrow, distal margin of telson concave with tuft of setae. G1 (Fig. 3K–P) slender, distally slightly curved toward dorsolateral side; tip chitinous, with truncated lobe in lateral view, curved toward dorsolateral side, two angles strongly prominent.

##### Description.

Carapace (Figs 2A, C, 3A) subquadrate, 1.1–1.2 × (*n* = 127) as wide as long; dorsal surface flat, finely punctate, glabrous; regions weakly defined, with noticeable groove between epigastric regions; hepatic region smooth, without small granules; metabranchial region sloping outwards. Front broad, slightly convex near orbital regions; frontal margin concave medially, lined with small, rounded granules, slightly divided into two indistinct lobes; postfrontal region distinct, separated into two lobes by shallow grooves.

Supraorbital margins lined with small granules. Anterolateral margin with three teeth including external orbital tooth; first tooth (external orbital tooth) largest, broad, bluntly triangular, slightly sloping forward; second tooth distinct, bluntly triangular; third tooth smallest, sometimes small but distinct or indicated by a small notch in small individuals. Posterolateral margins convergent posteriorly, covered with dense setae; posterolateral regions covered with dense setae near margins. Infraorbital ridge consisting of several small, rounded granules. Epistome broad, median part triangular.

Third maxillipeds (Figs 2B, D, 3B) broad, external surface almost glabrous; merus with shallow oblique groove along mesial margin of external surface, anterolateral angle broadly rounded, sloping laterally; ischium with an indistinct shallow groove slightly mesial to midline of external surface; exopod broad, convex, ~ 1.3 × as wide as ischium.

Chelipeds (Fig. 2) symmetrical in male and female, typically larger in male. Inner angle of carpus (Fig. 3C) without spines in male (with small distinct spine in female; Fig. 3D). Inner surface of palm glabrous; in male (Fig. 3E), proximal 1/2 of fingers with tufts of long dense soft setae on outer surface, slightly extending to palm side; base of fingers with pulvinus in male; in female (Fig. 3F), outer surface of palm glabrous and slightly granulated, inner surface glabrous; immovable finger with ridge consisting of small granules toward palm, fingers with short setae at tips; base of fingers without pulvinus.

Ambulatory legs (Figs 2, 3G, H, 5A) slender, P3 and P4 almost equal in length, longest, P5 shortest; setation not different between male and female. Meri without spines, but with long soft setae on proximal 1/2–2/3 of anterior margin; posterior margins almost glabrous; dorsal surfaces almost glabrous. P2 (Fig. 2) relatively short; propodus covered with dense short setae on anterior margin and ventral surface; dorsal surface glabrous, anterior margin of distal half densely covered with short setae; dactylus covered with rows of short setae on margins and surface. P3 and P4 (Figs 2, 3G, 5A) relatively long; meri ~ 2.9 × as long as wide (holotype); carpi glabrous on margins and surfaces; propodi ~ 2.2 as long as wide (holotype), dorsal surface glabrous, with dense short and sparse long setae on distal 1/3–1/2 of posterior margin and distal anterior margin (denser on distal part); dactyli almost equal to propodi in length, with dense short setae on anterior and posterior margins. P5 (Figs 3H, 5A) relatively short; merus ~ 2.8 × as long as wide (holotype); carpus glabrous on margins and surfaces; propodus ~ 1.4 × as long as wide (holotype), dorsal surface glabrous, with dense short and sparse long setae on distal 1/3–1/2 of posterior margin and distal anterior margin (denser on distal part); dactylus with dense short setae on almost entire anterior and posterior margin, denser on anterior margin.

Male pleon (Fig. 3I) narrow; external surface smooth, without any granules; lateral margins lined with short setae; telson subrectangular, significantly longer and narrower than sixth pleonal somite, distal margin concave, with a tuft of setae. Female pleon (Fig. 3J) wide; external surface smooth, without any granules; lateral margins lined with short setae; telson bluntly triangular, almost equal to sixth pleonal somite in length.

G1 (Fig. 3K–P) slender, distally slightly curved toward dorsolateral side; tip chitinous, with truncated lobe in lateral view, curved toward dorsolateral side, two angles strongly prominent; G2 shorter than 1/4 length of G1.

Female vulvae (Fig. 3Q) with one elongated short and one rounded sternal vulvar covers; sunken on mesial part.

##### Color in life.

The coloration of this new species is highly variable, so color is not a reliable characteristic for identification. The body color often resembles the color of the habitat substrate. Common body colors include dark brown or grayish black (Fig. 2E) or ivory white (Fig. 2F), with occasional individuals appearing in pale brown or orange (Fig. 2G, H). The carapace frequently has scattered spots, and the ambulatory legs often exhibit banding (e.g., Fig. 2E–G). The preserved specimens examined are pale brown.

##### Ecology.

This species primarily inhabits estuaries or freshwater downstream areas influenced by tide, often sheltering under stones or pebbles in shallow waters or near the water. Its habitat is diverse, including sandy bottoms, coral sand, and rocky shores. *Ptychognathusdajie* sp. nov. frequently coexists with other *Ptychognathus* species in similar habitat environments. In Taiwan, this species has been observed to be sympatric with several species, such as *P.insolitus*, *P.ishii*, *P.lipkei*, *P.sakaii*, *P.stimpsoni*, and *P.takahasii*.

##### Size.

Largest male 20.2 × 16.8 mm (NCHUZOOL 16074); largest female 16.8 × 14.5 mm (NCHUZOOL 17336); smallest ovig. female 7.7 × 7.0 mm (NCHUZOOL 17342).

##### Distribution.

Malaysia (Tioman) (type locality), Japan (Kochi, Ehime, Kanagawa, Izu Islands, and Ryukyu Islands), Taiwan, China (Guangdong), the Philippines (Negros, Cebu, and Camiguin), Indonesia (Sumatra, Ternate, and Bali Island); and Thailand (Phuket) (De Man 1898, 1902; Dai and Yang 1991; Ng 2006; Sasaki 2023; present study) (Fig. 1).

##### Etymology.

The species is named in honor of the late Dr. Ngan Kee Ng, who focused on this varunid group as part of her doctoral research. The specific epithet *dajie*, in Chinese, is a form of address to a senior woman in a leadership or caretaker role, similar to “big sister” or “senior sister”. Used as a noun in apposition.

##### Remarks.

*Ptychognathusdajie* sp. nov. is morphologically similar to a group of species within *Ptychognathus*, viz. *P.barbatus*, *P.glaber*, *P.guijulugani*, *P.ngankeeae* De Mazancourt, Mazel, Marquet, Poupin & Keith, 2024, *P.pusillus*, *P.sakaii*, and *P.stimpsoni*, in the following features: small body size, subquadrate carapace, and ambulatory legs without densely long setae. Nonetheless, the new species can be distinguished from these congeners primarily by the shape of the male telson and the pattern of setation in the ambulatory legs and male telson. The morphology of the male telson in *P.dajie* sp. nov., in particular, is rather unique in the genus in that the distal margin of male telson is obviously concave and with a distinct tuft of setae (Figs 2B, 3I) (vs neither concave nor truncated and without tufts of setae in other species). There is, however, no obvious interspecific difference in female telsons. Moreover, based on the unique morphology of the male telson in this species, Hsu and Shih (2020) also recognized it as distinct from *P.barbatus* and referred to it as P.aff.barbatus.

*Ptychognathusdajie* sp. nov. can be further differentiated from other congeners by the form of the carapace and the setation of the ambulatory legs. In *P.dajie* sp. nov., there are three obvious anterolateral teeth (including the external orbital tooth) on the carapace, and the second tooth is distinct and bluntly triangular (Fig. 3A) (vs single tooth and 1 or 2 notches or weak teeth in *P.glaber* and *P.sakaii*; Komai et al. 2021: figs 1A, 2A; Hsu et al. 2022: figs 1A, 2A).

**Figure 4.**
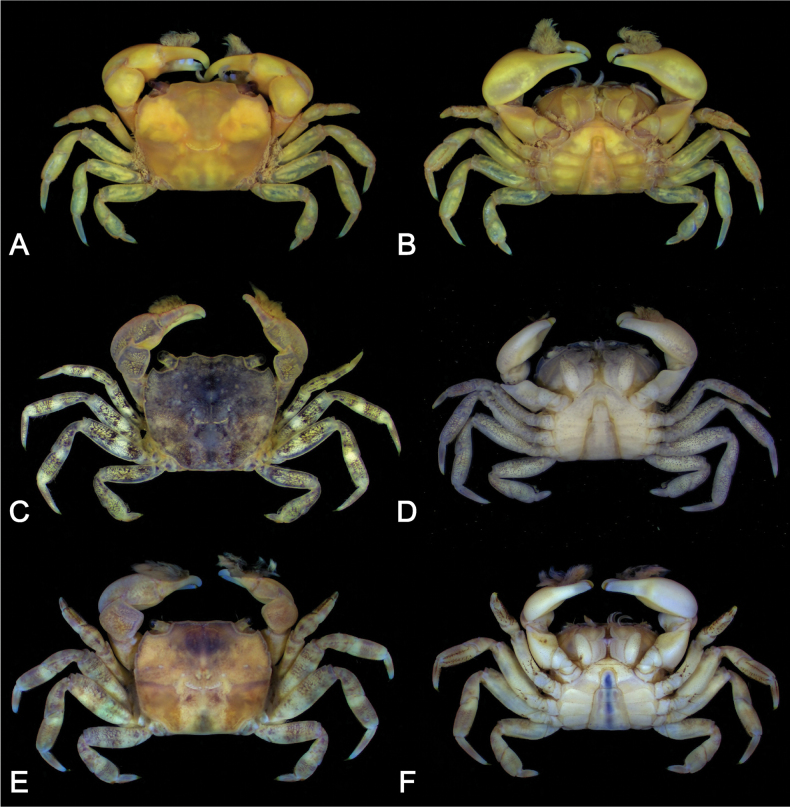
Three species of *Ptychognathus*. **A, B.***Ptychognathusbarbatus* (A. Milne-Edwards, 1873) (male, CW 10.2 mm, ZRC 2019.1806); **C, D.***Ptychognathusguijulugani* Rathbun, 1914 (male, CW 8.8 mm, NCHUZOOL 17334); **E, F.***Ptychognathuspusillus* Heller, 1865 (male, CW 13.2 mm, ZRC 2013.1844). **A, C, E.** Dorsal view; **B, D, F.** Ventral view.

**Figure 5.**
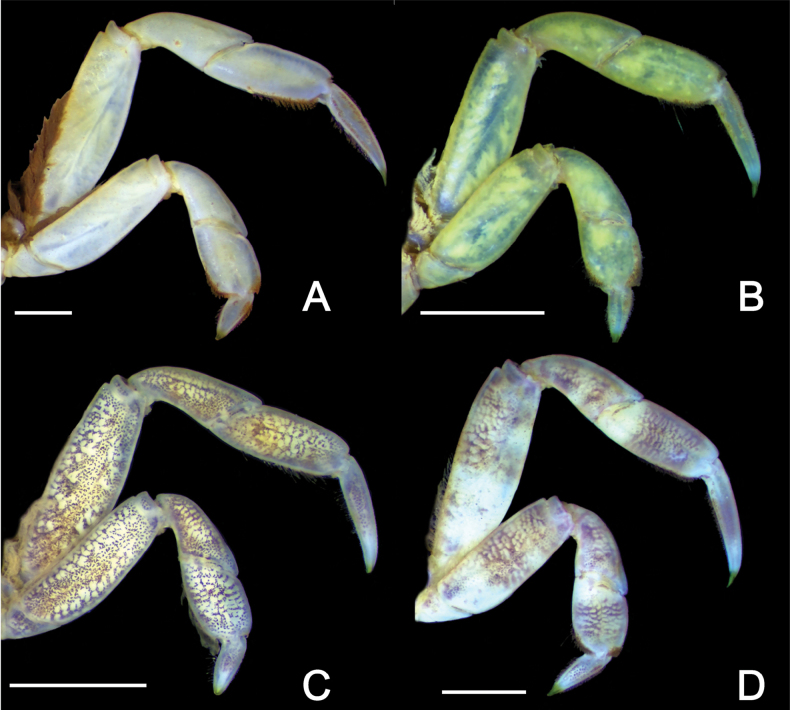
The right P4 and P5 of four species of *Ptychognathus* (dorsal view). **A.***P.dajie* sp. nov. (holotype male, CW 13.2 mm, ZRC 2024.0072); **B.***Ptychognathusbarbatus* (male, CW 10.2 mm, ZRC 2019.1806); **C.***Ptychognathusguijulugani* (male, CW 8.8 mm, NCHUZOOL 17334); **D.***Ptychognathuspusillus* (male, CW 13.2 mm, ZRC 2013.1844). Scale bars: 3 mm.

**Figure 6.**
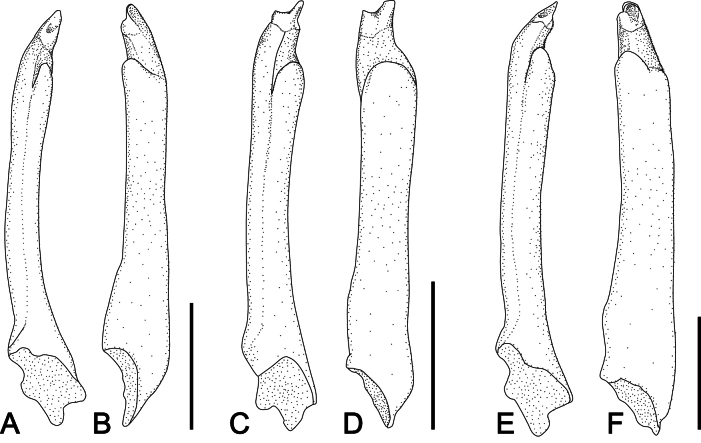
The right G1s of three species of *Ptychognathus*. **A, B.***Ptychognathusbarbatus* (male, CW 10.2 mm, ZRC 2019.1806); **C, D.***Ptychognathusguijulugani* (male, CW 8.8 mm, NCHUZOOL 17334); **E, F.***Ptychognathuspusillus* (male, CW 13.2 mm, ZRC 2013.1844). **A, C, E.** Dorsal view; **B, D, F.** Lateral view. Scale bars: 1 mm.

Although *P.dajie* sp. nov. and four other closely allied species (*P.barbatus*, *P.guijulugani*, *P.pusillus*, and *P.stimpsoni*) all have a similar carapace, including shape and anterolateral teeth morphology, they can be distinguished by the setation of the ambulatory legs. In *P.stimpsoni*, the surface near the anterior margins of the ambulatory carpi and propodi is covered with dense, short setae (Hsu and Shih 2020: figs 1C–F, 4G, H) (vs not covered with dense setae in *P.dajie*, *P.barbatus*, *P.guijulugani*, *P.ngankeeae*, and *P.pusillus*; Figs 2, 3G, H, 4, 5; see Tesch 1918: pl. 4, fig. 6; De Man 1905: pl. 17, fig. 1; De Mazancourt et al. 2024: figs 11E, F, 12A, C, 13E, F). There are five species within *Ptychognathus*, including *P.barbatus*, *P.guijulugani*, *P.ngankeeae*, *P.pusillus*, and *P.dajie* sp. nov., have very similar morphologies in the carapace, chelipeds, and shape of ambulatory legs; they can be discriminated by male pleons, the setation of ambulatory legs, and G1s (Table 2). In *P.dajie* sp. nov., as mentioned above, the distal margin of male telson is distinctly concave, with a distinct tuft of setae (Figs 2B, 3I) (vs not concave and without distinct tuft of setae in other four species; Fig. 4B, D, F; see De Mazancourt et al. 2024: fig. 13G). In term of the setation of ambulatory legs, there are some differences between these five species: a) in *P.dajie* sp. nov., the anterior margin of the P4 propodus bears setae only on the distal part, while the rest of the margins are almost glabrous (Figs 2, 3G, 5A) [vs almost whole anterior margin covered with setae, denser in distal part in *P.barbatus*; Figs 4A, B, 5B; see De Mazancourt et al. 2024: fig. 11E); the anterior margins are almost glabrous in *P.guijulugani*, *P.ngankeeae*, and *P.pusillus* (Figs 4C–F, 5C, D; see De Mazancourt et al. 2024: fig. 13E)]; b) in three species (*P.dajie* sp. nov., *P.ngankeeae* and *P.pusillus*), the P5 propodi bear short setae on distal 1/3–1/2 of posterior margins, and distal anterior margins (Figs 2, 3H, 4E, F, 5A, D; see De Mazancourt et al. 2024: fig. 13F) [vs with short setae on distal 1/3–1/2 of posterior margin, and distal 1/2–2/3 anterior margin in *P.barbatus* (Figs 4A, B, 5B; see De Mazancourt et al. 2024: fig. 11F); with dense long setae on distal 1/2–2/3 of posterior margin, and with short setae on distal anterior margin in *P.guijulugani* (Figs 4C, D, 5C)]. In addition, the chitinous structure of G1 are also different: in *P.dajie* sp. nov., the chitinous structure is a truncated lobe in lateral view, and the two angles are strongly prominent (Fig. 3K–P) [vs chitinous structure bluntly rounded, angles not prominent in *P.barbatus*, *P.ngankeeae*, and *P.pusillus*; Fig. 6A, B, E, F; see De Mazancourt et al. 2024: figs 11I, 13H); chitinous structure truncated, two angles slightly prominent in *P.guijulugani* (Fig. 6C, D)].

**Table 2.** Comparison of characters of five species of *Ptychognathus*.

Character	*P.dajie* sp. nov.	*P.barbatus*	*P.guijulugani*	*P.ngankeeae*	*P.pusillus*
Male telson (distal margin)	concave, with distinct tuft of setae (Figs 2B, 3I, I’)	not concave, without tufts of setae (Fig. 4B)	not concave, without tufts of setae (Fig. 4D)	not concave, without tufts of setae (De Mazancourt et al. 2024: fig. 13G)	not concave, without tufts of setae (Fig. 4F)
P4 anterior margin	setae only on the distal part, rest almost glabrous (Figs 3G, 5A)	setae on almost whole margin, denser in distal part (Fig. 5B)	almost glabrous (Fig. 5C)	almost glabrous (De Mazancourt et al. 2024: fig. 13E)	almost glabrous (Fig. 5D)
P5 anterior margin	setae only on distal part (Figs 3H, 5A)	setae on distal 1/3–1/2 (Fig. 5B)	setae only on distal part (Fig. 5C)	setae only on distal part (De Mazancourt et al. 2024: fig. 13F)	setae only on distal part (Fig. 5D)
P5 posterior margin	short setae (Figs 3H, 5A)	short setae (Fig. 5B)	dense long setae (Fig. 5C)	short setae (De Mazancourt et al. 2024: fig. 13F)	short setae (Fig. 5D)
G1 (chitinous structure)	truncated, angles strongly prominent (Fig. 3K–P)	rounded, angles not prominent (Fig. 6A, B)	truncated, angles slightly prominent (Fig. 6C, D)	rounded, angles not prominent (De Mazancourt et al. 2024: fig. 13H)	rounded, angles not prominent (Fig. 6E, F)

### ﻿DNA analyses

**Figure 7.**
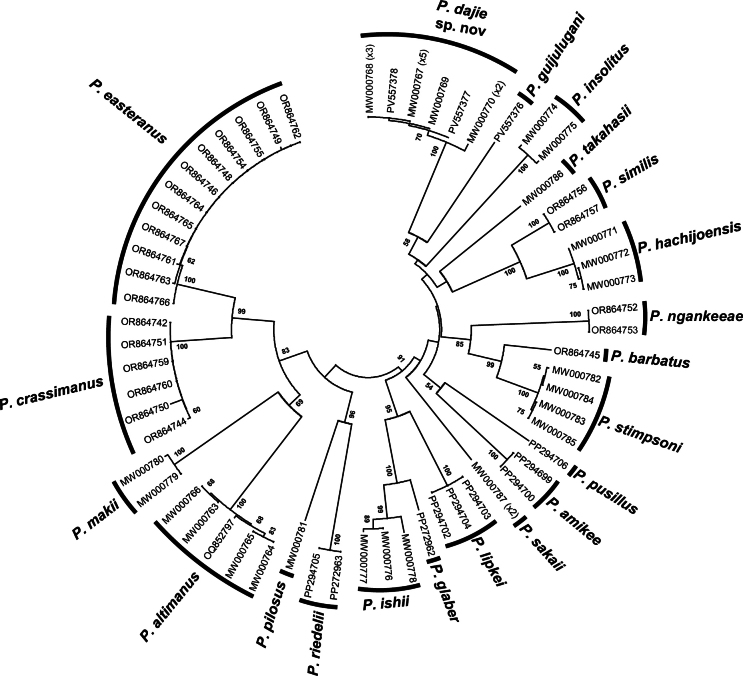
A neighbor-joining tree for species of *Ptychognathus*, based on the COI gene. Probability values at the nodes represent support values. Only values > 50% are shown. For haplotype names, see Table 1.

The NJ tree identified 21 OTUs (operational taxonomic units) with high support, corresponding to the new species in present study and 20 other previously described species (Fig. 7). The pairwise nucleotide divergences of K2P distances and bp differences of haplotypes between the new species and the 11 other species are shown in Table 3. The maximum intraspecific diversity of *P.dajie* sp. nov. is 0.77% (5 bp); while the interspecific divergences are 11.89–15.81% (71–102 bp) between *P.dajie* sp. nov. and the other species with similar morphologies (Table 3). The minimum interspecific distance (11.89%, 71 bp, between *P.dajie* sp. nov. and *P.pusillus*) is higher than those of several taxa of intertidal crabs, including some genera in Varunidae, such as *Pseudohelice* Sakai, Türkay & Yang, 2006 (1.54%; Prema et al. 2022), *Helice* De Haan, 1833 (excluding *H.latimera* complex, 2.97%; Ng et al. 2018), *Parahelice* Sakai, Türkay & Yang, 2006 (excluding between *P.r.* sp. 1 and *P.r.* sp. 2, 8.2%; Hsu and Shih 2024c), *Parapyxidognathus* Ward, 1941 (10.17%; Hsu and Shih 2024b), and some other genera in other families, such as *Parasesarma* De Man, 1895 (0.92%, Sesarmidae Dana, 1851; Shih et al. 2023), *Tuerkayana* Guinot, NK Ng & Rodríguez Moreno, 2018 (1.23%, Gecarcinidae MacLeay, 1838; Ng and Shih 2023), *Minuca* Bott, 1954 (4.61%, Ocypodidae Rafinesque, 1815; Thurman et al. 2023), and *Macromedaeus* Ward, 1942 (9.7%, Xanthidae MacLeay, 1838; Yuan et al. 2022).

**Table 3.** Matrix of percentage pairwise nucleotide divergence using Kimura 2-parameter (K2P) distances and the mean numbers of base pair differences based on the cytochrome c oxidase subunit I (COI) between *P.dajie* sp. nov. and selected related species of *Ptychognathus* (see Table 1, Fig. 7). The range of values is shown in parentheses.

	*P.guijulugani*	*P.pusillus*	*P.amikee*	*P.hachijoensis*	*P.barbatus*	*P.stimpsoni*	*P.sakaii*	*P.takahasii*	*P.insolitus*	*P.similis*	*P.ngankeeae*
Nucleotide divergence	12.08	11.93	13.18	14.82	14.51	13.05	12.95	14.19	15.67	15.36	15.58
	(11.89–12.27)	(11.89–12.08)	(12.98–13.37)	(14.16–14.98)	(14.33–14.53)	(12.7–13.65)	(12.78–12.97)	(14.03–14.23)	(15.41–15.81)	(14.93–15.78)	(15.24–15.74)
bp difference	72	72.3	78.5	85.9	85	78.8	77	84.1	91.2	85.7	89
	(71–73)	(71–82)	(77–83)	(82–87)	(84–88)	(76–88)	(76–78)	(83–87)	(90–92)	(76–91)	(83–102)

Among all *Ptychognathus* species, based on the constructed phylogenetic tree and calculated genetic distances, *P.dajie* sp. nov. is most closely related to *P.guijulugani* and *P.pusillus* (Fig. 7, Table 3). These three species also share similar characters, such as the structure of the carapace (Figs 2, 3A, 4C–F) and ambulatory legs (Figs 3G, H, 5A, C, D). According to the NJ tree (Fig. 7), *P.guijulugani* is the sister species of *P.dajie* sp. nov., although the support value is not high, and its G1 morphology resembles that of *P.dajie* sp. nov. more closely than that of other *Ptychognathus* species (Figs 3K–P, 6C, D). These observations suggest that species with closer phylogenetic relationships in *Ptychognathus* tend to exhibit more similar morphologies.

## ﻿Discussion

Previous records of “*Ptychognathusbarbatus*” from East and Southeast Asia (e.g., De Man 1902; Sakai 1939; Dai and Yang 1991, etc.) have accompanying descriptions and/or images indicating that these specimens may actually represent *P.dajie* sp. nov. rather than *P.barbatus* s. str. De Man (1902: 505) noted that the telson in male specimens from Atjeh (Indonesia) was shorter with a rounded distal margin, whereas in specimens from Ternate (Indonesia), the telson was longer with a concave distal margin. Based on these telson characters, the specimens from Ternate should be *P.dajie* sp. nov., while the specimens from Atjeh described in De Man (1895, 1898, 1902) do not represent *P.dajie* sp. nov. (De Man 1902: 505). According to the distribution and morphology, it is inferred that De Man’s Atjeh specimens may correspond to *P.pusillus*. However, Ng (2006) examined certain Atjeh specimens (RMNH-D1228; Nationaal Natuurhistorisch Museum (formerly Rijksmuseum van Natuurlijke Historie), Leiden, The Netherlands) and confirmed that their telsons possess a concave distal margin, aligning with the morphology of *P.dajie* sp. nov., suggesting that *P.dajie* sp. nov. may also be present in Atjeh. Additionally, studies of Sakai (1939), Dai et al. (1986), Dai and Yang (1991), and Toyota et al. (2019) contain descriptions or images indicating that the male telson’s distal margin in specimens identified as *Ptychognathusbarbatus* is concave and bears a tuft of setae, which conforms to the morphology of *P.dajie* sp. nov.

## Supplementary Material

XML Treatment for Ptychognathus dajie

## ﻿Additional information

### Author ORCIDs

Jhih-Wei Hsu https://orcid.org/0000-0002-9353-6140

Jose Christopher E. Mendoza https://orcid.org/0000-0001-9729-5703

Hsi-Te Shih https://orcid.org/0000-0003-1317-8783

### Data availability

All of the data that support the findings of this study are available in the main text or Supplementary Information.
